# Prevalence and Etiology of Traumatic Injuries to the Anterior Teeth among 5 to 8 Years Old School Children in Mathura City, India: An Epidemiological Study

**DOI:** 10.5005/jp-journals-10005-1308

**Published:** 2015-09-11

**Authors:** Sushma Gojanur, Ramakrishna Yeluri, Autar Krishen Munshi

**Affiliations:** Senior Lecturer, Department of Pedodontics and Preventive Dentistry, KD Dental College and Hospital, Mathura, Uttar Pradesh, India; Professor, Department of Pedodontics and Preventive Dentistry, KD Dental College and Hospital, Mathura, Uttar Pradesh, India; Former Professor and Head, Department of Pedodontics, KD Dental College and Hospital Mathura, Uttar Pradesh, India

**Keywords:** Anterior teeth, Dental trauma, Epidemiology.

## Abstract

**Objectives:** To assess the prevalence of traumatic injuries to the anterior teeth among the 5 to 8 years old children attending the schools in Mathura city.

**Study design:** A total of 1657 children of the age groups: 5, 6, 7 and 8 years from 20 schools situated in various parts of Mathura city were included in this study, utilizing stratified cluster random sampling method.

**Results:** The prevalence of traumatic injuries to the anterior teeth in 5 to 8 years old age group was found to be 2.7%. Males accounted for 3.1% whereas females accounted for 2.3%. Overall, males experienced more traumatic injuries than the females with male to female ratio of 1.8:1. The etiology of traumatic injuries was mostly due to falls, followed by bicycle accidents, collisions, violence and bike accidents in that order.

**How to cite this article:** Gojanur S, Yeluri R, Munshi AK. Prevalence and Etiology of Traumatic Injuries to the Anterior Teeth among 5 to 8 Years Old School Children in Mathura City, India: An Epidemiological Study. Int J Clin Pediatr Dent 2015;8(3):172-175.

## INTRODUCTION

Traumatic injuries to the anterior teeth among the young children are tragic but often an ignored problem. Children with injuries to their anterior teeth, and their concerned parents present a challenge for the dentist that is often unparalleled. There is perhaps no single dental disturbance that has a greater psychological impact on both the parents and the child than the fracture or loss of a child’s anterior tooth, especially if the injury involves an extensive loss of the tooth structure.^1^ Traumatic injuries, being on the rise, are the third largest cause for the mortality of teeth. With the so-called advanced civilized modes of teaching, children are more exposed to situations where trauma becomes a mandatory consequence of involvement. During the school age, children actively indulge in outdoor play. Though these activities are markers of growth and development of the child, careless activities, loss of balance and impaired movements increase the possibility of injuries. Traumatic dental injuries in the primary dentition are related to possible sequelae affecting the permanent succedaneous teeth and malformation has been estimated to occur in 25 to 69% of cases.^2^

Uttar Pradesh is the most populous state of the country (166.52 million in 2001), with 70 districts as per the census 2001. Mathura district has an area of 3,329.40 sq. kms and its population was 20.70 lakhs in 2001. Mathura city is under Mathura block. According to 2001 census, Mathura has 31 wards with total population of 3.23 lakhs.^3^ Although Mathura region has many hospitals and a dental college, the epidemiological data regarding the prevalence of traumatic injuries, which is very essential to formulate an action plan to combat them was unavailable in the literature. Hence, this study was conducted to assess the prevalence of traumatic injuries to the anterior teeth amongst the school-going children of Mathura city in the age group of 5 to 8 years.

## MATERIALS AND METHODS

In totality, 1657 children of the age groups: 5 to 8 years from 20 schools situated in various parts of Mathura city were included in this study. The study population was selected by stratified cluster random sampling methodology. The sample size was determined using the following formula:^4^

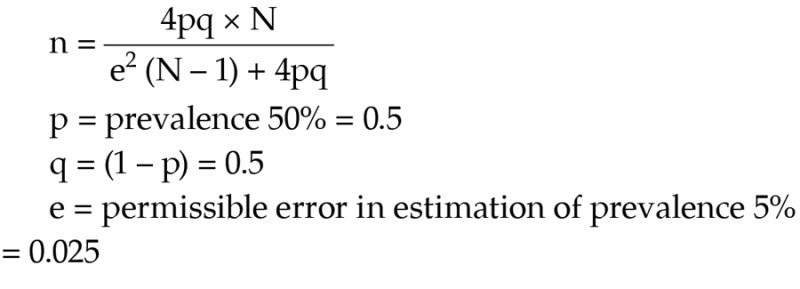



N = study population 10,690

n = sample size

A preliminary visit was made to these schools in order to obtain consent from the respective school authorities. The children were seated in an ordinary chair that was positioned to ensure adequate day light to facilitate the examination. A proforma was prepared to collect the data about the general information which was obtained from the school records and traumatic injuries. The traumatic injuries were classified according to the Ellis and Daveys classification.^5^ The instruments used were: plane mouth mirror, periodontal probes which conform to World Health Organization (WHO) specifications, containers (one for used instruments and one for sterilizing instruments), concentrated sterilizing solution and gauze.^6^ Current national recommendations and standards were followed for infection control and waste disposal.

## STATISTICAL ANALYS

All the collected data were entered in the Microsoft excel sheet 2007 version and subjected to statistical analysis using SPSS software version 11.5. Independent ‘t-test’ was used for comparison between males and females. The significance for all the statistical tests was predetermined at p < 0.05 (5%).

## RESULTS

The prevalence of traumatic injuries to the anterior teeth in 5 to 8 years old age group was found to be 2.7%. The 5 years old children (3.6%) presented with the largest number of injuries followed by 6, 8 and 7 years old (3.4, 2.4 and 1.4%, respectively). Males accounted for 29 (3.1%) affected children in the study, whereas females accounted for 16 (2.3%). Overall, males experienced more traumatic injuries than the females with male to female ratio of 1.8:1. Table 1 shows the distribution of study sample according to the age and gender. Tables 2 and 3 show prevalence of traumatic injuries to anterior teeth according to age and gender, respectively.

Regarding the type of trauma according to the Ellis and Davey’s classification, it was observed that the class IX fracture was the most frequent type in which 35 teeth were affected, followed by classes I and II fractures.

**Table 1:** **Table 1:** Distribution of the study sample according to the age and gender

		*Male*				*Female*				*Total*			
*Age in years*		*No.*		*%*		*No.*		*%*		*No.*		*%*	
5		235		59.6		159		40.4		394		23.7	
6		268		57.9		195		42.1		463		28.0	
7		219		52.4		199		47.6		418		25.2	
8		224		58.6		158		41.4		382		23.1	
Total		946		57.1		711		42.9		1657		100	

**Table 2:** **Table 2:** Prevalence of the traumatic injuries to anterior teeth according to age

		*Total*					
*Age in years*		*No.*			*%*		
5		14			3.6		
6		16			3.4		
7		6			1.4		
8		9			2.4		
Total		45			2.7		

**Table 3:** **Table 3:** Prevalence of the traumatic injuries to anterior teeth according to gender

		*Male*				*Female*			
*Age in years*		*No.*		*%*		*No.*		*%*	
5		11		4.7		3		1.9	
6		7		2.6		9		4.6	
7		4		1.8		2		1.0	
8		7		3.1		2		1.3	
Total		29		3.1		16		2.3	

**Graph 1:**
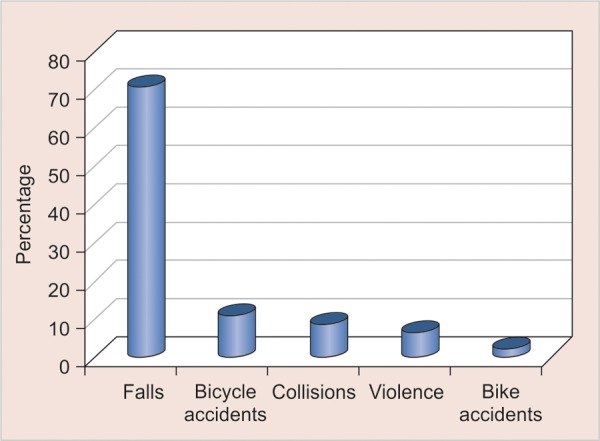
Prevalence of the traumatic injuries to anterior teeth according to cause of trauma

The teeth most commonly affected by dental trauma were the maxillary central incisors followed by maxillary lateral incisors. The most common cause of dental trauma in this study was due to falls (71.1%) followed by bicycle accidents (11.1%), collisions (8.9%), violence (6.7%) and bike accidents (2.2%). Graph 1 shows prevalence of traumatic injuries to anterior teeth according to cause of dental trauma.

## DISCUSSION

Physical activity is a basic need for the growth of a child. World Health Organization (WHO) theme for the year 2002 says ‘Move for health’, which emphasizes on the role of physical activity in the healthy living of an individual. During these physical activities, injuries to the face are one of the risks associated with it.^7^ Trauma to both primary and permanent dentition continues as a frequent dental problem. Trauma to the child dentition is an important issue, since fracture of one or more teeth, especially the anterior, may result in pain, loss of function, poor esthetics and psychological trauma.^8^ Traumatic dental injuries constitute a true dental emergency and require immediate assessment and management.

The prevalence of traumatic dental injuries worldwide ranges from 6 to 37%.^9-14^ The prevalence of traumatic injuries to the anterior teeth in 5 to 8 years old age group was found to be 2.7% in the present study. The 5-year-old children (3.6%) presented with the largest number of injuries followed by 6, 8 and 7 years old (3.4, 2.4 and 1.4%, respectively). A lower prevalence of the traumatic injuries was observed in the present study as compared with other studies (10-35%).^8,15-18^ Rai and Munshi (1998) observed a prevalence of 5.29% among 3 to 16 years old school going children in South Kanara.^19^ Gupta et al in 2002 found a prevalence of 39.26% in 8 to 10 years old children in south Kanara.^20^ In Jordan, a prevalence of 10.5 to 19.2% has been reported for trauma to anterior teeth.^10,21^ Federico and Garcia-Godoy^22^ in 1984 found that prevalence of traumatic injuries was 10.0% in Santo Domigo in 5 to 14 years old children.^22^

In the present study, 5 years old children (3.6%) presented with the largest number of injuries followed by 6, 8 and 7 years old (3.4, 2.4 and 1.4%, respectively). However, Othman et al showed that the most commonly affected age group was the 8 to 10 years old group and this was in agreement with some studies.^11,12,23^

Males accounted for 29 (3.1°%) affected children in the present study, whereas females accounted for 16 (2.3%). Overall, males experienced more traumatic injuries than the females with male to female ratio of 1.8:1. The general agreement in the literature about male predominance of dental trauma has been reported in the majority of the previous studies,^8,10-12,14,24^ which were consistent with our findings. Gupta et al showed a male to female ratio of 2:1 in south Kanara district.^20^ This might be related to their tendency of being more energetic and choosing more active and vigorous games and outdoor games with higher trauma risk than girls. However, there is one study in Jordan which reported that there were no gender differences.^25^

Regarding the type of trauma according to the Ellis and Davey’s classification, it was observed that the class IX fracture was the most frequent type of fracture followed by classes I and II fractures.^5^ The most common type of injury in permanent teeth was enamel fracture followed by enamel and dentin fracture. This was similar to the study conducted by Hunter et al in 1990, Delattre et al in 1994 and Marcenes et al in 1999.^26-28^

The findings of this study, such as boys experience dental trauma more frequently than the girls and the most affected teeth are the maxillary incisors, corroborate the findings of Sanchez et al, Hunter et al, Caliskan et al, Borssen et al, Oikarinen et al and Sane et al, with the exception of Garcia-Godoy et al who found a sex ratio of 0.91:1^8,16,20,22,26,29-31^

The most common etiologic reasons are falls, automobile and bicycle accidents, collisions and sporting activities. Our study revealed that the etiology of traumatic injuries was mostly due to falls (71.1%), followed by bicycle accidents (11.1%), collisions (8.9%), violence (6.7%) and bike accidents (2.2%); as was also found in a study conducted by Al-Jundi where falls caused 80% of dental injuries.^24^ This finding complies with most of the studies in the literature.^14,28,32^

In contrast, the rate of collisions, traffic accidents, sports, and fights were the least common causes. The study conducted by Marcenes et al in 1999 showed that most reported cause of trauma was violence (42.5%), followed by traffic accidents (24.1%).^28^ Gupta et al in 2000 reported that fractures occurred most commonly at home, followed by school and road accidents.^20^ The study conducted by Federico et al in 1984 showed most common cause of injury was falling against an object (78%), followed by struck by an object (14.6%).^22^

## CONCLUSION

- The prevalence of traumatic injuries to the anterior teeth in 5 to 8 years old age group was found to be 2.7% in the present study. The 5-year-old children (3.6%) presented with the largest number of injuries followed by 6, 8 and 7 years old (3.4, 2.4 and 1.4%, respectively).
- Males accounted for 29 (3.1%) affected children in the present study, whereas females accounted for 16 (2.3%). Overall, males experienced more traumatic injuries than the females, with male to female ratio of 1.8:1.
- Our study revealed that the etiology of traumatic injuries was mostly due to falls (71.1%), followed by bicycle accidents (11.1%), collisions (8.9%), violence (6.7%) and bike accidents (2.2%) in that order.
